# Sustainable Leadership, Environmental Turbulence, Resilience, and Employees' Wellbeing in SMEs

**DOI:** 10.3389/fpsyg.2022.939389

**Published:** 2022-06-28

**Authors:** Qaisar Iqbal, Katarzyna Piwowar-Sulej

**Affiliations:** ^1^Centre for China-India-Pakistan Studies, Sichuan University of Science and Engineering, Zigong, China; ^2^Department of Labor, Capital and Innovation, Faculty of Management, Wroclaw University of Economics, Wrocław, Poland

**Keywords:** sustainable development, market turbulence, workplace wellbeing, wellbeing at work, developing country

## Abstract

Drawing on the conservation of resources theory and contingency theories of leadership, this study aims to investigate how sustainable leadership (SL) influences employees' wellbeing (WB) through employee resilience (ER) and to examine the moderating effect of environmental turbulence (ET) on the “sustainable leadership-employees' wellbeing” relationship. Data were collected from 593 employees and 373 supervisors adopting two-wave design among small and medium-sized enterprises (SMEs) in China. The authors used structural equation modeling to empirically test the hypothesized model in this study. The research shows that SL is significantly related to the employees' WB in SMEs. Regarding mediating effect, SL also indirectly influences employees' WB through ER. Moreover, the impact of SL on employees' WB becomes more prominent in the presence of lower ET. To the best of the authors' knowledge, no prior study is available about the integrated relationship of SL, ER, ET, and employee WB.

## Introduction

Managing sustainability is one of the most challenging and rapidly growing areas in organizations. Sustainable development, which is based on three pillars, i.e., social, economic, and environmental, evokes the need of sustainable leadership (SL) (Slimane, 2012; Iqbal and Piwowar-Sulej, 2022; Xuecheng et al., 2022). Sustainable leaders can “allow a fast, resilient response which is competitive and appealing to all stakeholders” (Gerard et al., 2017, p. 116). One of the companies' stakeholders is an employee. Since securing healthy lives and promoting wellbeing (WB) is one of the sustainable development goals (United Nations, 2016), sustainable leaders should focus on the promotion of employee WB. The latter can be defined as an individual's experience of health, happiness, and prosperity (and efficiency (Sahai and Mahapatra, 2020). Subjective WB can be viewed as a phenomenon which covers hedonic (feeling good) aspect whereas psychological WB covers eudaimonic (functioning well) aspects (Aked et al., 2008; Fisher, 2014; Zacher and Rudolph, 2021).

Previous studies proved a strong positive relationship between the subjective WB of employees and their workplace performance (Trudel-Fitzgerald et al., 2019; Altman, 2021). The higher level of WB at workplace will lead to high self-esteem, meaningful participation, more positive workplace relationships, and control over one's own career and life (Kun and Gadanecz, 2019). This will result in higher employee productivity and efficiency (Sahai and Mahapatra, 2020).

Achievement of the above-presented positive outcomes depends on the level of employee WB. In turn, individual work-related WB depends on both job features and non-job features (Bennett et al., 2017) as well as internal (personal) and external factors (Oskrochi et al., 2018).

One of the internal factors—a valuable framework which needs further exploration (Harms et al., 2018)—is employee resilience (ER) referring to the individual's capacity for resourceful and flexible interaction with stressors (Klohnen, 1996). In turn, external factors are associated with various stressors which have a strong negative impact on WB. The mechanism of how and when stress occurs is explained by the conservation of resources theory (COR). This theory emphasizes the role of leader in preventing resource depletion and supporting staff in collecting resources which will have a positive impact on the employees' WB (Hobfoll et al., 2018). Since sustainable leaders focus, i.e., on social needs, they should directly positively influence employees' WB. Moreover, they may also develop employees' resilience through non-instrumental approach to employees, empowering them, matching actions with given words, making justice (Salehzadeh, 2019; Wang et al., 2019), and in this indirect way contributing to employees' WB.

As far as external factors influencing WB are concerned, it is worth highlighting that contemporary organizations operate in a dynamic environment. Companies have to face growing competition requiring innovation, technological disruptions, climate, social, political changes, and other emergencies (van Fenema and Romme, 2020). This environmental turbulence (ET) may negatively impact employees' WB which is also emphasized in the COR (Lanivich, 2015). Furthermore, taking into account the contingency theories of leadership (CTL), one can state that SL cannot always be the best way of managing people, especially in rapid changing circumstances (Wolinski, 2010). Therefore, the moderating influence of ET on the relationship between SL and employees' WB is worth empirical exploration.

Although there are studies linking leadership with ER and employees' WB (Nguyen et al., 2016; Salehzadeh, 2019; Zhu et al., 2019), none of them explored the linkage between the SL and work-related WB. This constitutes a promising field to be explored. Moreover, research examining ways in which WB can be reliably increased and the mechanisms underlying the relationship between leadership and work-related WB is still not enough developed (Page and Vella-Brodrick, 2009; Arnold, 2017). ET may be proposed as additional factors that could further explain the path from SL to WB as postulated by Li and Tong (2021).

The purpose of this article is to theoretically and empirically explore the relationships between SL and employees' subjective WB, including such mediating factor as ER and such moderating factor as ET with the use of assumptions of the COR and CTL. This paper reports on the results obtained based on a survey method. Considering highly competitive and market oriented situation (Ren and Chadee, 2017), increasing level of stress among workforce (Zou et al., 2021), and the lack of evidence from the perspective of employees' WB (Zhou et al., 2020), empirical research was conducted among employees working in small and medium-sized enterprises (SMEs) in China.

Implementation of SL in SMEs is important because SMEs have a significant contribution to the development of employment globally and sustained economic growth (Holt and Powell, 2015; Strauss et al., 2017). In China, they create ~80% of jobs (Zeng et al., 2014). About 20 years ago, the practices related to health promotion were rarely taken up in SMEs, although today, even international programs are held to promote WB in such enterprises (De Angelis et al., 2020). Previous research in SMEs examined employees' WB in the context of absenteeism (Holt and Powell, 2015; Rind et al., 2020) and determined the antecedents of WB (Maziriri et al., 2019) and the level of psychological WB (Zeng et al., 2014). The latter turned out to be much worse as compared to that of large firms (Luo et al., 2012; Zhou et al., 2020). In China, the work environment is highly competitive and market-oriented (Ren and Chadee, 2017); therefore, negative emotions among employees are rising perpetually (Yang et al., 2019). In a survey, Chinese Health Education Centre concluded that 50% of employees in their sample of population are suffering with stress and depression (Zou et al., 2021). As stated in the literature (Zeng et al., 2014; Yang et al., 2019), employees' WB in SMEs in China has been largely neglected so far both in academia and practice. As above-presented, the level of employees' WB in SMEs is lower than in large firms and this justifies a need to conduct further research on this topic. Furthermore, publications encompassing SL are still in their infancy (Gerard et al., 2017; Iqbal et al., 2020b). As far as SMEs are concerned, SL was theoretically modeled (Kerr, 2006) and empirically examined in, e.g., Thailand (Suriyankietkaew and Avery, 2016) and Pakistan (Iqbal et al., 2021). Although it was proved in the circumstances of SMEs' functioning that the leadership style impacts employee satisfaction (Suriyankietkaew and Avery, 2016), satisfaction is only one of the dimension of employees' WB (Ruggeri et al., 2020) and not always predicts a high job performance.

The above highlights the research gap to be filled by this study. Moreover, this paper contributes to the development of science not only through theoretical and empirical exploration of the relationships between SL, ER, and employees' WB but also through examination of both possible mediating and moderating mechanisms between SL and employees' WB. It also contributes to empirical studies which are based on the COR and CTL. Matching these theories, in turn, allows for better explanation of complex phenomena than the use of only one theory. Moreover, the present research contributes methodologically to literature by collecting data from multi sources, i.e., employees and their supervisors with 1-month time interval.

The remaining parts of this article are organized as follows. In the second section, a literature background is presented in terms of theories used in this study and relationships between examined variables. This leads to hypotheses formulation. In the third part of this paper, the materials and methods are presented. Then, the results, followed by their implications, are discussed. Finally, the article ends with conclusions and limitations, and at the same time presents directions for further research.

## Literature Background and Hypotheses Development

### Theoretical Framework

As mentioned in the Introduction, the way of how a leader may impact employees' WB may be explained with the use of the COR. Stress—which negatively impacts employees' WB—may occur when people (a) lose their resources, (b) are threatened with resource loss, or (c) fail to win resources after investments into resources (Hobfoll, 2010). The literature distinguishes the following groups of resources: object resources (e.g., computer), personal resources (e.g., competencies and personal characteristics), condition resources (e.g., employment), and energy resources (e.g., money, knowledge) (Hobfoll et al., 2018). The main assumption in the analyzed theory is that increasing of individual resources is crucial for building individual's strength and thus WB (Di Fabio, 2017).

The COR emphasizes that individuals need social support, because other people may offer them resources they need, save them from stressful situations, or strengthen their depleting resources. In turn, this allows them to either recover resources or open the possibility of utilizing them. Fisher (2014) highlighted that employees' WB is the outcome of social interactions as the key element of an employee's positive experiences at work. However, developing a supportive social network—which is emphasized in the COR—requires a significant, long-term investment of resources to construct and maintain relationships of support (Cangiano et al., 2019). In this situation, a leader can become helpful by means of removing barriers to prevent resource depletion and supporting staff in collecting resources, thus avoiding needless stressing agents and boosting both employees' WB and performance (Hobfoll et al., 2018).

This study also uses the contingency theories of leadership (CTL). Although many theories argue which leadership style is best suited for improving individual, team, and organizational performance, the CTL focuses on how specific circumstances affect a leader's effectiveness. Different CTLs present diverse approaches toward the extent of subjective preferences influencing successful leadership. For example, Fiedler's contingency theory states that the individual preferences of a leader have an unquestionable impact on managing various situations successfully (Fiedler, 1967). This author stated that, from the “path-goal” theoretical viewpoint, favorable situations are best managed by human-oriented leaders, whereas unfavorable ones by those task-oriented. A symmetry is required between personal inclinations and situational circumstances (Seyranian, 2009). As Wolinski (2010) stated, the effectiveness of a leader is contingent on how well their leadership style suits specific circumstances. Taking the above into account, the question arises, if SL is effective leadership style in the situation of ET in terms of its impact on the employees' WB.

### Sustainable Leadership and Employees' Wellbeing

Sustainable leadership—being the focus of this study—is an emerging type of leadership which contributes to the organizational performance in the context of current and future environmental, economic, and social goals (McCann and Holt, 2010). Based on literature studies (e.g., Gerard et al., 2017; Burawat, 2019; Iqbal et al., 2020a), one may state that the concept of SL is based on the assumptions of transformational leadership (Waldman et al., 2006), as well as ethical leadership (Wu et al., 2015), responsible leadership (Maak and Pless, 2006), positive leadership (Salmi et al., 2014), reflexive, and participative leadership (Gerard et al., 2017). Sustainable leaders, such as transformational leaders, stimulate and inspire followers focusing on their needs, and—like participative leaders—involve employees in decision-making. They use positive behaviors as a method to guide others which is in line with positive leadership. They also, similarly to responsible leaders, take into account the interests of all company stakeholders and represent high ethical values to achieve a common goal—such as ethical leaders. Moreover, they are thoughtful like reflexive leaders.

Many authors (e.g., Tafvelin et al., 2011; Kara et al., 2013; Arnold, 2017) emphasized that transformational leadership is a leadership style which positively influences employee WB. As far as the COR is concerned, transformational leaders strengthen subordinates' personal resources, reduce job demands, motivate subordinates to use resources efficiently, and provide job resources (Diebig et al., 2017; Harms et al., 2017; Berger et al., 2019). Ethical, responsible, and positive leadership also effectively stimulates employees' WB (Kelloway et al., 2013; Bardoel et al., 2014; Chughtai et al., 2015; Haque, 2021).

Sustainable leadership has an impacts on many resources. It enhances knowledge sharing, development of employees, participation, and empowerment (Avery and Bergsteiner, 2011; Gjerde and Ladegård, 2019; Iqbal and Ahmad, 2020). SL covers shared responsibility to preserve economic and human resources as far as avoid environmental and social degradation (Hargreaves and Fink, 2006). Finally, sustainable leaders construct positive narratives in organizational context leading to increase in subordinates' energy (Di Fabio, 2017) which is one of personal resources. Therefore, the following hypothesis was formulated:

*H1: There is a positive association between sustainable leadership and employees' wellbeing*.

### Employee Resilience as Mediator

As Southwick et al. (2014) stated, the definition of resilience requires identifying whether it is approached as an attribute, an action or an effect, which frequently results in resorting to a dual perspective when specifying either presence or absence of resilience. The synonym of resilience is ego-resiliency (Block and Kremen, 1996). Initially, this phenomenon was referred to as stress resistance (Masten and Tellegen, 2012; Hartmann et al., 2021). In this study, resilience is treated as an individual ability “that allows the person to find and use internal and external resources to overcome adversities or regain equilibrium once they have occurred” (Djourova et al., 2020, p. 4).

Many authors have emphasized the importance of resilience for an individual positive functioning in society (Alessandri et al., 2012; Gao et al., 2017). As far as professional life is concerned, ER helps employees to accept work circumstances and find meaning in difficulties (Coutu, 2002; Linnenluecke, 2017) and increases their adaptation to changes (Lengnick-Hall et al., 2011) as well as their recovering from failures (Shin et al., 2012). Dias Abreu and Rodriguez Blanco (2017) found that WB at work is positively correlated with ER. ER was, e.g., used as a mediator between workplace humor and workplace WB (Bhattacharyya et al., 2019).

As indicated before, based on the assumptions of the COR, leaders may increase employees' WB through securing their resources. Leadership style is one of situational antecedent of ER as well (Harland et al., 2005; Wang et al., 2019), being often linked with emotion management (Thiel et al., 2015; Richard, 2020). Resilience can be treated from the COR's lens as an important personal resource (Smith et al., 2010; Shin et al., 2012; Wang et al., 2019). It was empirically proved that leader-guided, downregulation of negative emotions increases ER (Young et al., 2017; Pollock et al., 2020). Transformational leadership which is based on, i.e., charisma, intellectual stimulation, inspirational motivation, and individualized consideration, is positively associated with ER (Harland et al., 2005; Djourova et al., 2020) whereas a passive leadership decreases ER (Wang et al., 2019). There is also a positive relationship between empowering leadership, making justice and matching actions with given words and ER (Seibert et al., 2011; Nguyen et al., 2016; Salehzadeh, 2019). Transformational leadership training was used to enhance the resilience in teams (Molenaar, 2010) before. In the literature, it is also assumed that the moral responsibility of a leader is to generate ER, especially in the time of crisis (Välikangas, 2020).

As presented in Section Theoretical Framework, SL is a socially, economically, and environmentally-oriented leadership style. It promotes employees' empowerment and is based, i.e., on the transformational leadership style. Previous research has also identified mediating effect of ER between self-efficacy (as the outcome of transformational leadership) and workplace WB (Djourova et al., 2020). Given the positive effect of ER on employees' WB and the impact of transformational leadership style on resilience, the latter factor is also expected to positively mediate the relationship between SL and employees' WB. The assumption is reflected in the following hypothesis:

*H2: Employees' resilience positively mediates the relationship between sustainable leadership and employees' wellbeing*.

### Moderating Role of Environmental Turbulence

Contemporary organizations function in an increasingly complex environment (Davila and Elvira, 2012). Technological, economic, and social circumstances may all shift unexpectedly and rapidly, which in consequence place new constraints on the behavior and achievements of people and their organizations (Boyne and Meier, 2009). ET (rapid changes in the external company's environment), characterized by ambiguity and uncertainty, play a crucial role in determining the sustainability of resource systems (Agrawal, 2001), which leads to stress (Iannello et al., 2017), anxiety (Waldman et al., 2001) and reducing WB (Hancock and Mattick, 2020).

While developing high-quality relationships with different stakeholders, sustainable leaders determine potential changes in the business environment (Gerard et al., 2017). In this way, they may secure necessary resources and maintain employees' WB. However, even if a supervisor acts along with SL patterns, the contingency theories of leadership states that a given leadership style is not always the most effective.

Previous research has proven that interorganizational change, as postulated in the literature response of leaders to ET (Pennings, 1992), generated internal turbulence, created destabilization and uncertainty (Boyne and Meier, 2009), decreased job security (Reilly et al., 1993), and thus negatively impacted WB. Moreover, transformational leadership style in the dynamic banking sector was found as contributing to high levels of workplace stress, which leads to work burnout (Parveen and Adeinat, 2019). In turn, Li and Tong (2021) stated that employees who face an uncertain environment prefer to rely on narcissistic leadership. They proved that the latter, i.e., energetic, self-confident, goal-directed, and dominant leadership style, has positive influence on ER in case of ET.

Based on the reasoning above, it is expected that SL cannot always be the best way of managing people, especially in rapid changing circumstances. The level of ET will moderate the effect of SL on employees' WB. Specifically, the effect of SL on employees' WB will be higher with low level of ET. The following—last—hypothesis is suggested for the moderating role of ET:

*H3: Environmental turbulence moderates the relationship between sustainable leadership and employees' wellbeing so that high environmental turbulence weakens their relationship*.

**Figure 1:**
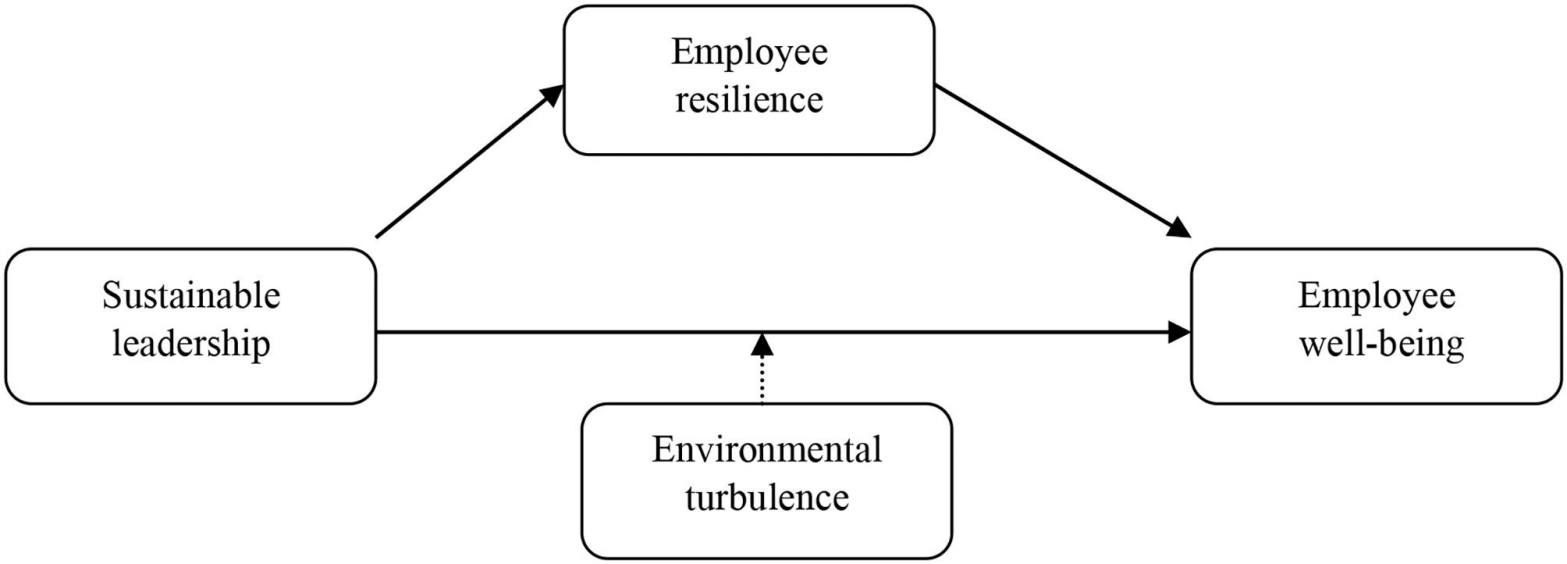
Research framework.

Based on the above literature review and hypothesis development, the following research framework is introduced (refer to Figure 1). In this framework, sustainable leadership (SL) is an independent variable and employee's wellbeing (WB) is a dependent variable. Employee's resilience (ER) has been introduced as a mediator. Moreover, environmental turbulence (ET) is a moderating variable.

## Research Methodology

This study has adopted a cross-sectional approach and gathered data from SMEs in China to test the research framework (Figure 1). Taking financial, networking, and time constraints into account while collecting data from a massive populations, the authors have adopted a cluster sampling approach to classify SMEs into different groups in relation to their geographical locations. Based on the simple random sampling approach, data have been collected from employees and their respective supervisors of SMEs in top five cities namely Shanghai, Beijing, Shenzhen, Guangzhou, and Chengdu of China. The authors have applied their personal and professional networking to collect data from 123 SMEs in China. They also shared cover letters with respondents to convey the objective of this research. Respondents were ensured about the confidentiality of their responses.

To cope with common method bias issues (Podsakoff, 2003), the authors adopted a two-wave design ensuring predictors and outcome variables at different events. They have sent 1,000 questionnaires to SME employees through their HR departments *via* email. In the first wave, employees were asked to report their perception about SL traits of their direct supervisors and to evaluate ET. In the first wave, 593 employees shared their responses with a response rate of 59.30%. Considering recommendations of De Lange et al. (2004) about the significance of time lags shorter than 1 year in the WB research, this study adopted 1-month time interval between two data collection waves. The authors have also used six digit codes to match responses during two waves. In the second wave, they have also taken support from the HR departments to recognize the concerned supervisors of employees and send them a survey form through an email. In the second wave, data were collected from direct supervisors of 593 employees who participated in the first wave. $\bar{\text{A}}$ total of 593 direct supervisors were asked to mark their perceptions about ER and employee WB. In the second wave, 379 supervisors provided their evaluation about their employees where response rate was 63.91%. A total of six survey forms were found incomplete and invalid. Ultimately, final 373 complete questionnaires were ready for further analysis in this study. The current dataset is different from time series data where data are collected from the same source over a period of time (Jebb et al., 2015). To examine minimum sample size required, this study has run G^*^Power application with 3 predictors, 0.15 effect size, and 0.80 significance power (Faul et al., 2009) which confirmed 77 as mandatory responses. Hence, 373 responses are good enough to provide valid empirical evidence in this study.

### Measures

The authors collected data through an online survey form which comprises of six sections, namely, SL, ET, ET, employee WB, and demographics. In this study, the survey form was prepared in English and translated into Chinese. This study adopted the back-translation process (Brislin, 1970) to examine the accuracy of the questionnaire. Furthermore, previous studies concluded with cognitive trouble and diminished quality issues with higher Likert scale (Robinson, 2018; Iqbal et al., 2021), so the authors measured constructs of this study based on a 5-point Likert scale ranging from strongly disagree to strongly agree.

To measure SL, this study has adopted 15-measurement items from the study of Iqbal and Ahmad (2020). This scale measures to which employees perceive about SL characteristics such as broad systems thinking, social and environmental consciousness, change orientation, business savvy, persuasiveness, adaptability, credibility, passion, energy, mentoring, and development as their supervisors exhibit. Sample item is “My supervisor's decisions are made while considering the entire organization.”

Environmental turbulence has been measured with 6-items adapted from the study of Wang and Fang (2012) and Wang et al. (2020). Employees rated about a level of uncertainty and unpredictability in their industrial environment from the perspective of both market and technological turbulence. A sample measurement items are “technology in the product development is rapidly changing” and “customer preferences are changing quite a bit over time.”

Employee resilience was measured with 6-items adopted from the study of Al-Hawari et al. (2020). Employees rated their resilience as a resource which negatively affects their interpersonal stressors and alleviates their emotional exhaustion. Sample item is “I feel I can handle many things at a time at my job.”

Considering the dual approach to WB (Ryan and Deci, 2001), which has been empirically supported (Slemp and Vella-Brodrick, 2014), it has been operationalized as comprised of both psychological WB and subjective WB in this study. A total of three items were adapted from the study of Keyes (2007) to measure subjective WB, and six items were taken to measure psychological WB (Slemp and Vella-Brodrick, 2014). Supervisors were asked to rate this measurement scale. Sample measurement items of psychological WB are “employees have experiences that challenge them to grow and become better people” and that for subjective WB is “employees feel interested in their life.”

Extant literature posits significant impact of demographics variables, namely, age, gender, and job tenure on the employees' WB (Torkelson and Muhonen, 2008; Muhonen et al., 2013; Padkapayeva et al., 2018). Moreover, irrespective of Chinese work values, Le et al. (2020) have also reported significant relationship of these demographic variables with employees' WB in China. Therefore, this study has taken into consideration of age, gender, and number of years in the firm as control variables. A pilot study was also conducted with a sample of 30 responses to assess the content validity of measurement items which concluded with no need for any amendments in the final survey form.

### Descriptive Analysis

In this study, demographic analysis through statistical program for social sciences (SPSS) revealed that there are 204 women (54.69%) and 169 men (45.31%) out of 373 employees. Most of the respondents out of the employees sample lie in the age category of 25–35 years old (36.19%) followed by age group of 36–45 years old (29.22%). A total of 186 out of 373 employees in this study have master qualification. Most of the respondents in this study have working experience of 11–15 years in current organization, i.e., (*n* = 151, 40.48%) followed by the group of 16–20 years (*n* = 86, 23.05%) Majority of the respondents (*n* = 112, 30.027%) have participated from Beijing, followed by respondents from Shanghai (*n* = 89, 23.861) whereas least respondents are from Guangzhou (*n* = 44, %11.796). Demographic analysis of supervisor sample revealed that they were on average of 43 years old. There were 63% male supervisor. Supervisors' tenure with the SME extended from 3 to 27 years. Mostly supervisors (*n* = 212, 57%) had master degree as their highest qualification and were serving at the level of senior manager (*n* = 145, 39%) followed by general manager (*n* = 85, 23%).

The authors have employed the criteria for mean values established by Sekaran and Bougie (2016) against 5-point Likert scale to evaluate the mean values of continuous variables in this study. In this study, there is high presence of ER (M = 4.203), and ET (M = 4.005) in SMEs in China. Yet, mean values indicate the low presence of psychological WB (M = 2.525), subjective WB (M = 2.869), and overall employees' WB (M = 2.640). SL has a moderate presence (M = 3.922) in Chinese SMEs (refer to Table 1).

**Table 1:** Descriptive statistics and normality of data.

**Construct**	**Mean**	**Std. deviation**	**Skewness**		**Kurtosis**	
	**Statistic**	**Statistic**	**Statistic**	**Std. error**	**Statistic**	**Std. error**
Employee resilience	4.203	0.579	−0.622	0.126	0.686	0.252
Sustainable leadership	3.922	0.596	−0.224	0.126	0.057	0.252
Environmental turbulence	4.005	0.608	−0.183	0.126	0.089	0.252
Employee wellbeing	2.640	0.394	−0.009	0.126	−0.630	0.252
Subjective wellbeing	2.869	0.482	−0.028	0.126	−1.033	0.252
Psychological wellbeing	2.525	0.429	0.001	0.126	−0.767	0.252
**Mardia's multivariate skewness and kurtosis**			**β**	***z***		***p*** **-value**
Skewness			1.97	122.607		0.000
Kurtosis			37.412	−10.434		0.000

### Data-Screening

Before data analysis, data-screening process was applied to examine the missing values, outliers, common method bias, and normal distribution. The mandatory marking against all measurement items in online survey form ensured the absence of missing values. The Z-score values of all cases were found <3.29, which indicates the absence of any univariate outliers. The authors have also run Mahalanobis distance test to assess any multivariate outlier in this study. The running of this test revealed one response as multivariate outlier, so deleted it prior to further analysis.

This study has also run adopted Harman's one factor test to statistically verify the absence of common method bias. Harman's one factor test revealed no indication of common method bias as first factor variance contributes only 44.07%. In this study, the authors have run the web application available at: http://webpower.psychstat.org/models/kurtosis/to assess the position of univariate and multivariate normality of data. Values of skewness and kurtosis for all continuous variables in this study are found in the range of −2 and +2, so there is univariate normality. Moreover, Mardia's skewness (β = 89.54, *p* < 0.01) and kurtosis values (β = 247.40, *p* < 0.01) show the absence of multivariate normality.

Furthermore, the authors conducted confirmatory factor analysis (CFA) in AMOS with maximum likelihood estimation because of normal distribution of data and free from multicollinearity issue (Kline, 2015). CFA revealed that goodness-of-fit indices possesses minor difference between actual and proposed model based on Kline (2015)'s criteria, i.e., RMSEA = 0.063 <0.07, CFI = 0.957 > 0.95, GFI = 0.959 > 0.95, and SRMR = 0.077 <0.08.

### Data Analysis

Being explanatory in nature and having complex framework which comprises both moderation and mediation, this study is suitable to employ partial least squares structural equation modeling (PLS-SEM) (Ringle et al., 2020). Partial least squares structural equation modeling (PLS-SEM) assesses both measurement model and structural model.

#### Measurement Model

Prior to path analysis, it is prerequisite to perform measurement model analysis that evaluates indicator reliability, internal consistency reliability, and construct validity for reflective constructs (Hair et al., 2020). In this study, all variables in the hypothesized model are reflective in nature.

**Figure 2:**
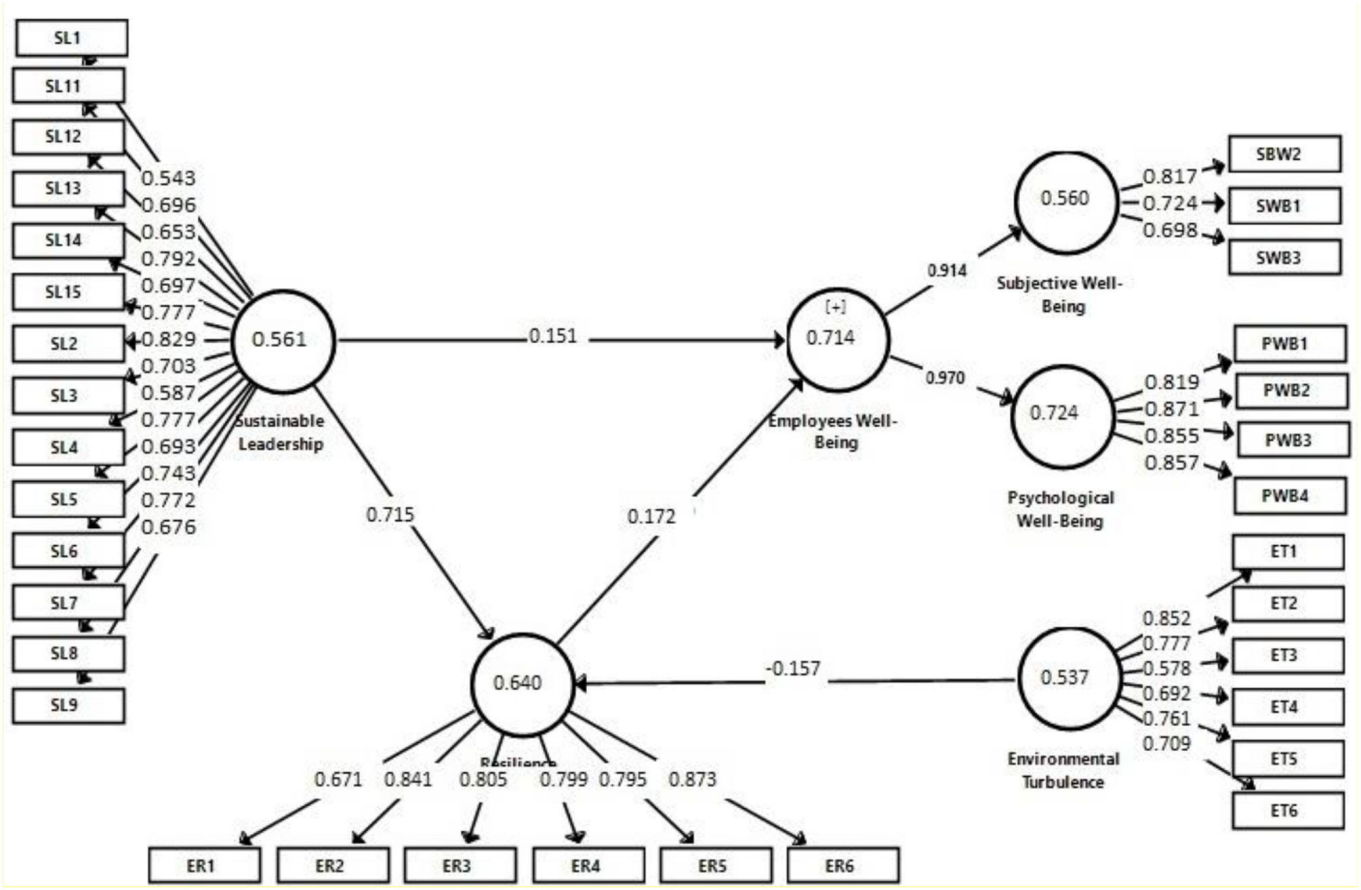
Measurement model analysis.

In this study, two items of psychological WB and one item of SL were deleted because of their factor loading below 0.50. The measurement analysis revealed that factor loading of rest of items ranged from 0.543 to 0.873 (refer to Figure 2). Cronbach's alpha values of all variables ranged from 0.749 to 0.897. Moreover, composite reliability values of all variables in this study extend from 0.791 to 0.935. In this study, average variance extracted values of all variables are found >0.50 (refer to Figure 2). Therefore, factor loading, Cronbach's alpha, composite reliability, and average variance extracted values are greater than their respective cutoff values. Based on the criteria of Hair et al. (2020), this study possesses significant convergent validity.

This study assessed discriminant validity using the Fornell–Larcker criterion (Henseler et al., 2015). As the square root of AVEs of all constructs in this study is greater than their correlations with other constructs (refer to Table 2), so there is acceptable discriminant validity in this study.

**Table 2:** Discriminant validity.

**Construct**	**1**	**2**	**3**	**4**	**5**	**6**
Employee resilience	**0.800**					
Sustainable leadership	0.729	**0.746**				
Environmental turbulence	−0.739	0.726	**0.733**			
Employee wellbeing	0.265	0.205	0.246	**0.845**		
Subjective wellbeing	0.336	0.233	0.289	0.782	**0.748**	
Psychological wellbeing	0.175	0.151	0.176	0.837	0.515	**0.851**

*The bold values indicates the square root of the respective AVE values*.

## Results

The analysis of structural model revealed that SL significantly influences employees' WB (β = 0.151, *p* < 0.05) in SMEs in China. Therefore, hypothesis H1 is accepted. Present empirical findings also indicated about significant indirect effect (β = 0.123, *p* < 0.05) of paths from SL to ER (β = 0.715) and that of ER to employees' WB (β = 0.172) (refer to Table 3). Therefore, hypothesis H2 is accepted in this study. Resultantly, SL substantially affects employees' WB through ER.

**Table 3:** Hypotheses testing.

**Hypothesis**	**B**	**S.D**.	***T*** **-value**	***p*** **-value**	**LLCI**	**ULCI**
SL > WB	0.151	0.028	5.412	0.000	0.096	0.206
SL > ER	0.715	0.032	21.767	0.000	0.650	0.779
ER > WB	0.172	0.043	3.974	0.000	0.087	0.257
SL > ER> WB	0.123	0.036	3.357	0.000	0.051	0.195
SL^*^ET > WB	−0.092	0.0411	−2.239	0.025	−0.173	−0.011

The ^*^ symbol indicates the interaction of SL with ET.

Current empirical evidence also indicates that the interaction term of SL and ET has significant negative impact of the employees' WB (β = −0.092, *p* < 0.05) in Chinese SMEs (refer to Table 3). Therefore, hypothesis H3 is supported in this study. Current empirical findings claim that ET significantly weakens the relationship of SL with employee WB. Higher levels of ET stifle the “SL-employee WB” relationship.

## Discussion

This study has proven a positive association between SL and employees' WB. This means—in the light of COR—that sustainable leaders are able to prevent employees' resource depletion and support subordinates in collecting resources (Hobfoll et al., 2018). As indicated in the theoretical part of this paper, previous research on the relationship between leadership style and employees' WB focused on transformational leadership (Tafvelin et al., 2011; Kara et al., 2013; Arnold, 2017; Diebig et al., 2017) as well as ethical, responsible, and positive leadership (Kelloway et al., 2013; Bardoel et al., 2014; Chughtai et al., 2015; Haque, 2021). This study shows that SL—built on the assumptions of the above-presented leadership styles—positively stimulates employees' WB. Since SL is a sustainability-oriented leadership style, it should contribute to the achievement of three types of goals: environmental, economic, and social. The latter covers the issue of employees' WB.

Moreover, SL may contribute to increasing employees' WB, i.e., through increasing ER. As presented research shows, ER positively mediates the relationship between SL and employees' WB. This means that ER is one of the resources crucial for securing WB and that sustainable leaders similar to transformational leaders (Harland et al., 2005; Djourova et al., 2020) positively stimulate ER.

Finally, ET weakens the relationship between SL and employees' WB. In the context of CTL, one may paraphrase a statement by Wolinski (2010) admitting that SL as a leadership style does not match a specific situation of ET. The above findings have both theoretical and practical implications.

### Theoretical Implications

This study fulfills a postulate by Arnold (2017) and Inceoglu et al. (2018) and contributes to the body of academic knowledge through exploring the mechanism on how leaders impact employees' WB. It utilizes the assumptions of COR and CTL. Moreover, this is the first study which explains the process by which SL—as emerging type of leadership—impact employees' WB. Finally, this study—also as first—examines the above-presented mechanism in SMEs in China.

Consistent with previous research on transformational leadership (e.g., Tafvelin et al., 2011; Kara et al., 2013; Arnold, 2017), this study confirms the direct impact of SL on employee's WB. This refers to both subjective and psychological WB. However, this study also uses ER as mediator between SL and employees' WB and demonstrates the presence of significant positive mediation of this variable. It increases the understanding of the relationship between SL and WB clarifying that this relationship may be both direct and indirect.

The findings of this study indicate that the relationship between SL and employees' WB is not only mediated by ER but also moderated by ET. The latter, however, has a negative impact on this relationship, which is in line with the findings obtained by Parveen and Adeinat (2019) in their study on transformational leadership. People operating in highly-turbulent environment need more dominant that servant leaders.

An increase in the level of ET is expected in many regions due to the “continual growth in unanticipated developments in technical and market sectors” (Omar, 2022, p. 1023). Such phenomena as the COVID-19 pandemic have also made the market more turbulent and simultaneously decreased employees' WB (Harju et al., 2021). Therefore, this study adds an argument to the discussion about the role of a leader in shaping employees' performance in changing and unpredictable setting within which companies have to operate.

The above-presented considerations advance the understanding of the theory of SL. Previous studies have found relationship between SL and organizational sustainable performance treated as a bundle of economic, environmental, and social goals (e.g., Iqbal et al., 2020a; Iqbal and Ahmad, 2021). However, recent literature has highlighted that the focus on the triple bottom line may negatively impact employees and that employee dimension should be added to the basic sustainability pillars (Bush, 2020). This article reveals the role of SL in achieving one of the detailed goals in the form of employees' WB, which strictly refers to employee dimension of sustainability.

As far as the methodological aspect of this study is concerned, it fulfills the rigor of quantitative empirical examination to understand the mechanism of how SL influences employees' WB. Researchers may follow the presented methodological guidelines to extend the current state of knowledge about the relationship between SL and employees' WB in organizations other than SMEs.

### Practical Implications

The findings from this study have several practical implications. First, since employees' WB is closely linked with job performance (Kundi et al., 2021) and thus accomplishing organizational sustainable development goals, there is a need for measuring the level of WB and introducing WB-oriented practices. Although a barrier for SMEs for implementing formal WB initiatives may be a budget constraints (Collingridge, 2020), managers should develop and implement SL. In this study, the presented low level of employees' WB in SMEs in China (M = 2.64)—which corresponds with general findings by the Chinese health education (Zou et al., 2021)—even more justifies the implementation of SL in the organizations under the study.

Organizations can promote SL practices in their domains by enhancing long-term commitment, sharing sustainability-related visions and sustainable development goals, and engaging all stakeholders (Iqbal and Ahmad, 2020). In turn, sustainable leaders should directly influence the level of employees' WB through, e.g., HR development (Gjerde and Ladegård, 2019) and indirectly through, e.g., empowering and making justice (Salehzadeh, 2019; Wang et al., 2019), which leads to higher ER. At this point, it is worth emphasizing that activities directed toward the prevention of employees' resources were more important that activities aiming at collecting new resources because according to COR, resource loss is disproportionately more salient than resource gain (Westman et al., 2015).

The presented considerations also add to our understanding of how ET interacts between SL and employees' WB. Given the revealed negative effect of ET, shareholders should monitor the level of both SL and ET. In the case of surveyed SMEs, this issue is especially important because SL has a moderate presence (M = 3.922) and the level of ET is quite high (M = 4.005). The latter corresponds with such literature characteristics of the Chinese work environment as a high competition and market orientation (Ren and Chadee, 2017).

Finally, this article also provides useful material for educational institutions. Its findings can bring a novelty into the HRM and leadership-related subjects as well as courses aiming at increasing students awareness in the area of sustainable development. Considering the amount of time spent at work across individuals' lifetimes, employees' WB is a powerful factor of general quality of life of a society (Weziak-Bialowolska et al., 2020).

## Conclusions and Limitations

This study is in line with “research aiming to improve quality of life by offering a solid framework for measuring and understanding employee WB” (Page and Vella-Brodrick, 2009, p. 455). It extends the research on relationship between leadership and employees' WB. It focuses on an emerging type of leadership—i.e., SL—and aids in understanding the mechanism by which sustainable leaders can achieve high employees' WB. In particular, it adapts COR and demonstrates that SL significantly influences employees' WB in SMEs. Moreover, SL has significant indirect impact on employees' WB through shaping ER. However, a high level of ET (as a moderator between SL and employee WB) may decrease employees' WB, which provide evidence that—as CTL emphasizes—SL does not match rapid changing situations.

This study—like others—has some limitations. However, acknowledgment of such limitations creates an opportunity to make recommendations for further research. First, considering the generalization issue, the forthcoming research is suggested to be conducted in other countries and cover also large companies. Second, this study was based on self-reported data which is limited by the fact that it rarely can be independently verified. Therefore, a mixed-method approach should be used in further studies. Moreover, both SL and ET may have a longitudinal effect. In turn, resources—which is emphasized in the COR—are changing and dynamic (Westman et al., 2015). Therefore, a longitudinal mixed-method research is suggested to provide much deeper insight. Finally, future research may apply other theories than the COR and CLT, e.g., the broaden-and-build theory which emphasizes the role of positive emotions in shaping WB.

## Data Availability Statement

The original contributions presented in the study are included in the article/supplementary material, further inquiries can be directed to the corresponding author.

## Author Contributions

All authors listed have made a substantial, direct, and intellectual contribution to the work and approved it for publication.

## Funding

The project was financed by the Ministry of Science and Higher Education in Poland under the program Regional Initiative of Excellence 2019–2022 Project Number 015/RID/2018/19 total funding amount 10 721 040,00 PLN.

## Conflict of Interest

The authors declare that the research was conducted in the absence of any commercial or financial relationships that could be construed as a potential conflict of interest.

## Publisher's Note

All claims expressed in this article are solely those of the authors and do not necessarily represent those of their affiliated organizations, or those of the publisher, the editors and the reviewers. Any product that may be evaluated in this article, or claim that may be made by its manufacturer, is not guaranteed or endorsed by the publisher.
